# Anthrone-Based Dummy Molecularly Imprinted PVDF Membrane for Monitoring Fluorene and Phenanthrene in River Water

**DOI:** 10.3390/molecules30183754

**Published:** 2025-09-16

**Authors:** Aria Pinandita, Nurrahmi Handayani, Muhammad Iqbal, Untung Triadhi, Rusnadi Rusnadi, Samitha Dewi Djajanti, Muhammad Bachri Amran, Muhammad Ali Zulfikar

**Affiliations:** 1 Doctoral Program of Chemistry, Faculty of Mathematics and Natural Sciences, Institut Teknologi Bandung, Jl. Ganesha 10, Bandung 40132, West Java, Indonesia; 30520306@mahasiswa.itb.ac.id; 2 Analytical Chemistry Research Group, Faculty of Mathematics and Natural Sciences, Institut Teknologi Bandung, Jl. Ganesha 10, Bandung 40132, West Java, Indonesia; nurrahmi.105@itb.ac.id (N.H.); m.iqbal@itb.ac.id (M.I.); untung@itb.ac.id (U.T.); rusnadi@itb.ac.id (R.R.); samitha@itb.ac.id (S.D.D.); amran@itb.ac.id (M.B.A.); 3 Research Center for Nanosciences and Nanotechnology, Institut Teknologi Bandung, CAS Building 1-3rd Floor, Jl. Ganesha 10, Bandung 40132, West Java, Indonesia

**Keywords:** dummy molecularly imprinted membrane, semi-interpenetrating polymer network (semi-IPN), PVDF membrane, fluorene, phenanthrene, polycyclic aromatic hydrocarbons (PAHs)

## Abstract

The anthrone-based dummy molecularly imprinted membrane (DIM) was successfully synthesized using a semi-interpenetrating polymer network (semi-IPN) approach for the selective recognition and adsorption of fluorene and phenanthrene in aqueous systems. Fourier-transform infrared spectroscopy (FTIR) confirmed the successful incorporation of functional groups, while scanning electron microscopy (SEM) revealed a uniform porous morphology favorable for analyte diffusion. Thermogravimetric analysis (TGA) demonstrated good thermal stability, and Brunauer–Emmett–Teller (BET) and Barrett–Joyner–Halenda (BJH) analyses indicated an enhanced surface area and mesoporous structure that supported improved adsorption performance. Adsorption isotherm studies revealed favorable adsorption behavior, with the maximum adsorption capacities of the DIM calculated to be 130.857 mg/g for fluorene and 453.030 mg/g for phenanthrene. The imprinting factors (IFs) were approximately 2.01 for fluorene and 2.17 for phenanthrene, confirming the successful imprinting effect. The recovery values achieved were 86.61% for fluorene and 92.40% for phenanthrene, demonstrating the efficiency and selectivity of the fabricated membrane. These results highlight the potential application of the anthrone-based DIM in the environmental monitoring of polycyclic aromatic hydrocarbons (PAHs).

## 1. Introduction

Polycyclic aromatic hydrocarbons (PAHs) are a class of hydrophobic organic pollutants that are widely present in aquatic environments as a result of anthropogenic activities such as fossil fuel combustion, industrial emissions, and urban runoff [1,2,3]. Among these compounds, fluorene and phenanthrene are low-molecular-weight PAHs that are frequently found in river and surface waters. Due to their chemical stability and lipophilicity, these compounds are non-biodegradable and tend to accumulate in sediments and aquatic organisms, contributing to long-term environmental contamination.

Both fluorene and phenanthrene are known for their toxic, mutagenic, and potentially carcinogenic effects, especially with chronic exposure even at trace concentrations [3]. Therefore, their monitoring in environmental water samples is crucial for assessing ecological risks and ensuring water safety. However, accurate detection of these PAHs is often challenged by complex sample matrices, which can interfere with analytical precision and reduce method sensitivity [4,5].

Molecularly imprinted polymers (MIPs) offer a powerful strategy for improving the selectivity of sample preparation techniques through the creation of synthetic recognition sites that are complementary in shape and functionality to the target analyte [6,7,8,9,10]. Despite their high affinity and selectivity, conventional MIPs synthesized using the original analytes as templates pose significant limitations. The use of toxic target molecules such as PAHs as template molecules during polymerization raises safety concerns, and residual template molecules entrapped within the polymer network may lead to template bleeding, causing false positives during analysis [11,12,13].

To overcome these challenges, the dummy template imprinting strategy has been developed. In this approach, a safe structural analog of the target analyte is used as a surrogate template during polymerization, thereby avoiding the direct use of toxic targets. This strategy effectively minimizes health and environmental risks, prevents template leakage, and at the same time preserves the specificity and recognition capability of the resulting MIP [11,12,13,14]. In this study, anthrone—a tricyclic compound structurally similar to fluorene and phenanthrene—was employed as a dummy template. The use of anthrone not only minimizes health and environmental risks during polymer synthesis but also prevents the problem of template leakage while preserving the molecular recognition capability of the MIP.

To further enhance the practical application of MIPs in environmental analysis, a dummy molecularly imprinted membrane (DIM) was developed through an in situ polymerization method based on a semi-interpenetrating polymer network (semi-IPN) [15,16]. In this approach, electrospun poly(vinylidene fluoride) (PVDF) membranes were immersed in a pre-polymerization mixture containing functional monomers, crosslinkers, initiators, and the dummy template. This method allows the imprinting polymer to form within and around the fibrous membrane structure without disrupting its morphology [17,18,19,20,21,22,23,24,25]. The resulting anthrone-based DIM combines the high surface area and mechanical stability of PVDF nanofibers with the selective recognition properties of the imprinted polymer, providing an efficient platform for the selective extraction of fluorene and phenanthrene from river water samples. This study presents the fabrication, characterization, and application of the anthrone-based DIM, offering a safe, selective, and robust alternative for the environmental monitoring of PAHs in aqueous matrices.

## 2. Results and Discussion

### 2.1. Selection of Anthrone as a Dummy Template

**Figure 1:**
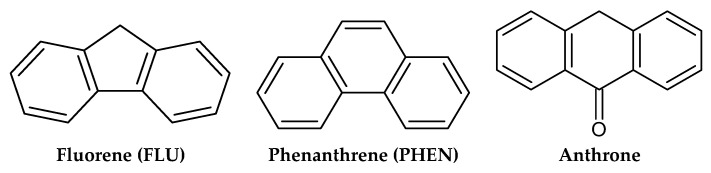
Molecular Structures of Fluorene, Phenanthrene, and Anthrone for Dummy Template.

The use of anthrone as a dummy template in the molecular imprinting process was primarily based on its structural similarity to the target analytes, fluorene and phenanthrene, both of which are PAHs. Structurally, anthrone consists of a tricyclic aromatic system with a six-membered ketonic ring fused to two additional benzene rings as depicted in Figure 1. This arrangement closely mimics the fused ring system of phenanthrene, which also possesses three aromatic rings, and fluorene, which contains a five-membered cyclopentadiene ring flanked by two benzene rings.

While the central ring in fluorene is a five-membered ring, the overall size, geometry, and electron distribution of anthrone still provide a sufficiently analogous spatial conformation to mimic the binding characteristics of both PAHs. The presence of the ketone group in anthrone introduces a polar functionality not found in fluorene or phenanthrene; however, this functionality can enhance the interaction with functional monomers (styrene) during imprinting, without significantly distorting the cavity structure.

Another key feature of anthrone is the presence of an sp^3^-hybridized carbon (C-sp^3^) adjacent to the ketone group. This non-planar center slightly disrupts the overall molecular planarity compared to fully aromatic systems like phenanthrene. However, this partial loss of planarity is advantageous for creating binding sites with sufficient flexibility, allowing the imprinted cavities to accommodate structurally similar PAHs that are not perfectly planar—such as fluorene, which contains a cyclopentadiene ring. This slight steric accommodation enhances the binding efficiency of the resulting polymer toward both fluorene and phenanthrene.

In addition to structural mimicry, safety considerations were a major factor in selecting anthrone. Fluorene and phenanthrene are known to be toxic, potentially carcinogenic, and environmentally hazardous. The use of these compounds as templates during polymerization may result in template bleeding, which not only compromises analytical accuracy but also poses risks to both human health and the environment. In contrast, anthrone is less toxic, more chemically stable, and easier to handle during synthesis, making it a safer and more practical alternative in dummy template molecular imprinting.

A computational chemistry approach based on electronic structure analysis was carried out to assess the similarity between the dummy template (anthrone) and the target analytes (fluorene and phenanthrene) in terms of frontier molecular orbitals and interaction energy [26,27,28,29,30,31,32,33]. As presented in Table 1 and Table 2, the HOMO–LUMO bandgap of the anthrone–styrene complex (6.2387 eV for the 1:1 ratio) lies between those of styrene–fluorene (6.5416 eV) and styrene–phenanthrene (5.9390 eV), indicating that anthrone provides an intermediate electronic environment that mimics both targets. Similarly, the binding energy (ΔE) of anthrone (−19.20 kJ/mol) is comparable to those of fluorene (−17.88 kJ/mol) and phenanthrene (−18.49 kJ/mol), supporting its suitability as a dummy template for imprinting both analytes.

**Figure 2:**
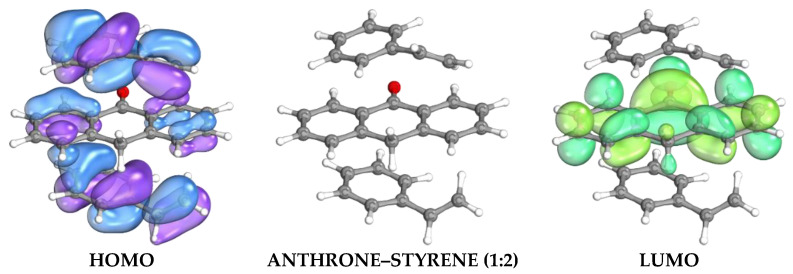
Visualization of HOMO–LUMO Electron Density Distributions for Styrene Complexe (1:2).

Complex stoichiometry optimization showed that the 1:2 anthrone-to-styrene ratio exhibited the most negative interaction energy (ΔE = −54.25 kJ/mol) with a slightly reduced bandgap (5.9441 eV), as presented in Figure 2, suggesting the most stable and favorable pre-polymerization complex. By contrast, the more positive ΔE obtained at the 1:3 ratio (−38.75 kJ/mol) indicates an energetically unfavorable configuration. Based on these computational results, the molar ratio of anthrone as the dummy template to styrene as the functional monomer was set at 1:2 for the synthesis. Therefore, the electronic structure and energy analysis substantiate the choice of anthrone as a dummy template and justify the 1:2 ratio for optimal imprinting performance, providing strong evidence for binding affinity and cavity compatibility.

### 2.2. Semi-Interpenetrating Polymer Network (Semi-IPN) Approach

The fabrication of the DIM was carried out using a semi-IPN strategy, which integrates two distinct polymer phases: the electrospun PVDF membrane as the supporting phase, and the molecularly imprinted polymer formed through in situ polymerization as the chemically crosslinked modifier [14,15]. During synthesis, the electrospun PVDF membrane was immersed in the pre-polymerization solution containing the dummy template, functional monomer, cross-linker, and initiator. This solution permeated the porous nanofiber network of the PVDF membrane, and subsequent thermal polymerization fixed the MIP within and around the fiber matrix. The PVDF nanofiber scaffold offers a high surface area, excellent chemical resistance, and hydrophobicity, making it an ideal platform to support the formation of imprinted cavities without compromising membrane integrity. Meanwhile, the pre-polymerization mixture acts as a functional modifier, embedding selective recognition sites directly into the PVDF matrix. This synergistic integration ensures that the resulting membrane retains both mechanical robustness and molecular selectivity, essential for use in aqueous environments.

### 2.3. Anthrone Template Removal from DIM

**Figure 3:**
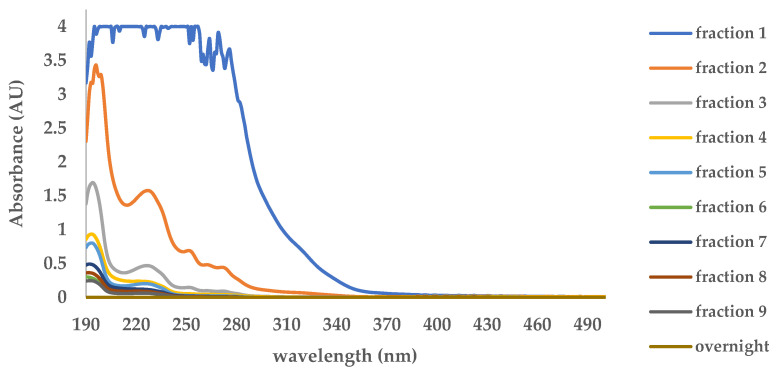
UV–Vis spectrophotometric monitoring of anthrone leaching from DIM.

UV-Vis spectroscopy was performed to monitor anthrone leaching in each washing fraction. As shown in Figure 3, the absorbance at 263 nm, corresponding to the maximum absorption wavelength (λmax) of anthrone, decreased markedly across sequential fractions and became undetectable after the tenth fraction (overnight washing). This confirms that template removal was complete, as no residual anthrone was present beyond this point. These results provide quantitative evidence supporting the successful elimination of the template from the prepared membrane. Furthermore, subsequent FTIR and TGA characterizations were carried out to corroborate the UV–Vis findings and provide additional confirmation of complete template removal.

### 2.4. FTIR Characterization

Fourier-transform infrared (FTIR) spectroscopy was used to characterize the functional groups present in anthrone, PVDF, non-imprinted membrane (NIM), DIM, and DIM after template molecule removal. Each material displayed distinctive absorption bands, allowing for interpretation of their chemical functionalities. For anthrone, a sharp absorption band was observed around 1680 cm^−1^, corresponding to the C=O stretching vibration of the ketone group. Additionally, bands in the range of 1500–1600 cm^−1^ were attributed to aromatic C=C stretching, while weak bands near 3000–3100 cm^−1^ were ascribed to aromatic C–H stretching, confirming its aromatic ketone structure.

In the FTIR spectrum of PVDF, prominent bands were detected around 1170 cm^−1^ and 1400 cm^−1^, which were assigned to C–F stretching vibrations, while the band near 840–880 cm^−1^ was related to CF_2_ wagging, characteristic of the PVDF backbone. No carbonyl or aromatic vibrations were identified, consistent with the non-aromatic, fluorinated polymer structure of PVDF. For the NIM sample, bands at approximately 1720–1730 cm^−1^ indicated the presence of carbonyl groups from monomers or cross-linkers (ethylene glycol dimethacrylate), while bands between 1150 and 1250 cm^−1^ were assigned to C–O–C stretching vibrations, indicating the formation of ester functionalities during polymerization [34,35].

The FTIR spectrum of DIM showed similar features but with additional peaks corresponding to the incorporated template. A carbonyl stretching band around 1680 cm^−1^, along with aromatic C=C bands in the 1500–1600 cm^−1^ range, confirmed the successful imprinting of anthrone. These characteristic bands were diminished in intensity in the spectrum of DIM after template removal, indicating that the anthrone template had been partially or completely removed from the polymer matrix.

**Figure 4:**
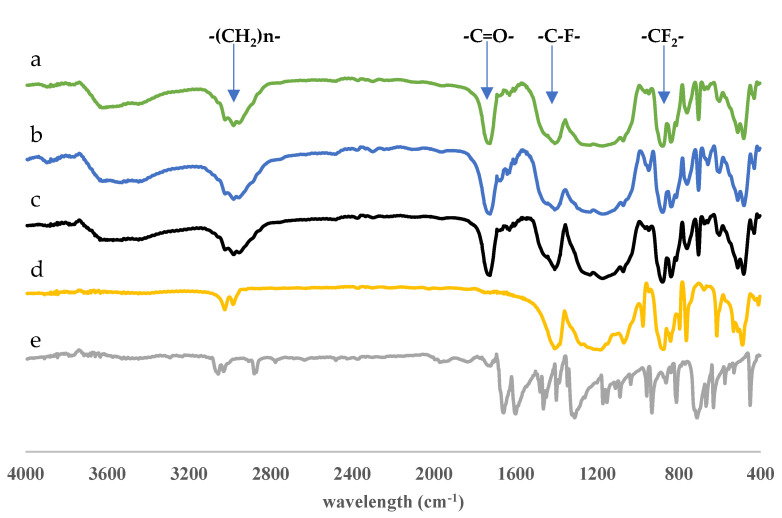
FTIR spectra of materials: (a) DIM after leaching, (b) DIM, (c) NIM, (d) PVDF membrane, and (e) anthrone template.

The comparison between DIM before and after leaching demonstrated a noticeable reduction in both carbonyl and aromatic absorption bands. This change suggested the successful template removal, leading to the formation of molecularly imprinted cavities within the polymer network. Meanwhile, the preservation of PVDF-related bands indicated that the PVDF support remained chemically stable throughout the synthesis and leaching processes as presented in Figure 4.

### 2.5. Thermal Analysis (TGA)

While FTIR spectroscopy provided valuable insights into the presence of functional groups within the composite materials, certain spectral regions exhibited potential overlap—particularly between the carbonyl group of anthrone and that of the cross-linker (ethylene glycol dimethacrylate), as well as between the aromatic C=C vibrations of anthrone and styrene (functional monomer). These overlaps may obscure definitive confirmation of complete template removal based solely on infrared analysis. Therefore, to complement the FTIR findings and provide further verification of anthrone elimination from the DIM matrix, thermal analysis using thermogravimetric analysis (TGA) and derivative thermogravimetry (DTG) was conducted. This approach allowed for the observation of characteristic thermal degradation patterns associated with the presence or absence of the template molecule.

**Figure 5:**
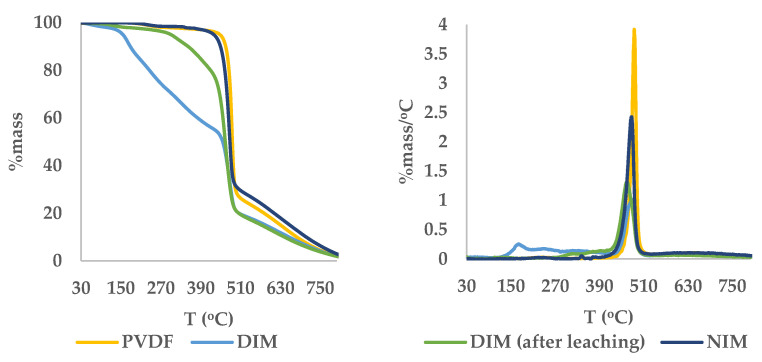
Thermogravimetric (TGA) and Derivative Thermogravimetric (DTG) Curves of DIM.

Thermal analysis using TGA-DTG was conducted to evaluate the thermal stability and confirm the efficiency of the leaching process for removing the dummy template anthrone from the DIM matrix as depicted in Figure 5. The DTG profile of DIM before leaching exhibited two distinct degradation stages. The first stage showed a DTG peak at 171.3 °C, which corresponds to the decomposition of residual anthrone trapped in the polymer network. This temperature aligns closely with the melting point of anthrone (154–157 °C), confirming its presence. The second degradation stage appeared at 479.4 °C, indicating the breakdown of the main polymer backbone composed of PVDF, styrene and EGDMA.

After leaching, the DTG profile of the DIM displayed only a single degradation peak at 464.9 °C, with the earlier anthrone-related peak no longer observed. This confirms the effective removal of anthrone. Additionally, the DTG peak profiles after leaching suggested a more defined degradation behavior due to the absence of anthrone. The thermal degradation pattern of NIM also showed a single peak at 477.3 °C, further confirming that the leaching process successfully rendered the DIM structurally similar to NIM in terms of thermal response.

The changes in degradation behavior after leaching also reflect structural alterations in the DIM. The removal of anthrone is believed to create specific recognition cavities, resulting in an increase in porosity and the formation of active binding sites. These structural changes may contribute to altered thermal stability. To support these findings, SEM and BET analyses were performed to confirm the formation of porous morphology and enhanced surface area in the DIM after leaching.

### 2.6. Morphological and Surface Characterization

**Figure 6:**
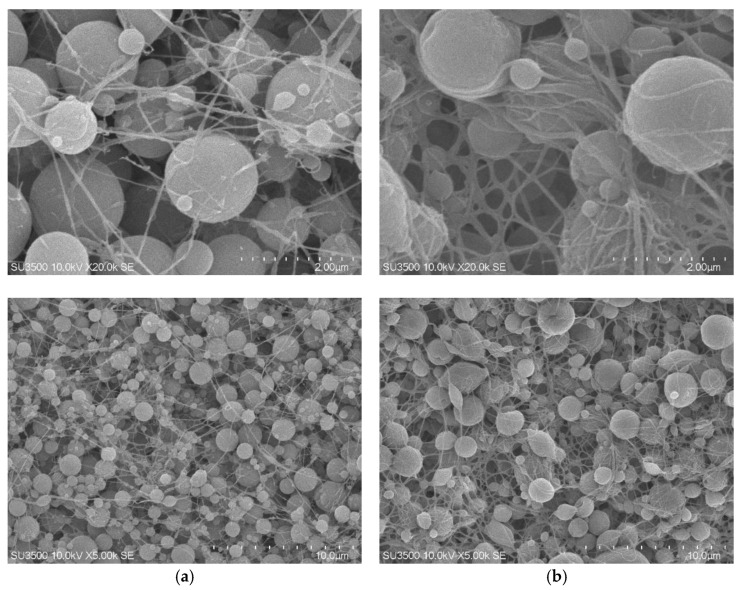
SEM Micrographs of DIM (**a**) and NIM (**b**).

The surface morphology of the synthesized polymers was characterized using scanning electron microscopy (SEM), revealing notable differences between the DIM and NIM as presented in Figure 6. The surface morphology of the DIM was observed to consist of uniformly distributed spherical particles with relatively homogeneous sizes. A fibrous network was also detected, forming interconnections between the particles and creating a more open porous structure. At higher magnifications, the presence of rougher surfaces was identified, which indicated the successful formation of imprinted cavities after the removal of the dummy template. In contrast, the NIM was characterized by a denser structure with spherical particles of more varied sizes. The fibrous network was observed to be less pronounced, leading to a lower degree of porosity compared to DIM. The surface appeared smoother and lacked visible cavities, suggesting the absence of molecular recognition sites.

Further characterization using BET surface area analysis revealed a significant difference in surface area between the two materials, as shown in Table 3. DIM exhibited a much higher specific surface area (4.662 m^2^/g) compared to NIP (0.447 m^2^/g), indicating a greater surface exposure resulting from the presence of imprinted cavities. Although the BJH desorption analysis showed that DIM had a lower pore radius (18.521 nm) than NIM (34.237 nm), the DIM exhibited a slightly larger pore volume (0.045 cc/g versus 0.015 cc/g for NIM), which is consistent with the presence of mesoporous structures formed during template extraction.

These combined results confirm that the template removal process successfully produced porous and morphologically distinct DIM particles with potentially higher adsorption capacities and selectivity due to the created cavities. To further validate the imprinting efficiency and the accessibility of the active cavities on the DIM surface, adsorption isotherm experiments were subsequently performed. These studies provide quantitative insight into the binding characteristics of the imprinted sites and serve as a critical confirmation of the selective sorption behavior of the DIM toward the target analyte.

### 2.7. Adsorption Isotherm Study

The adsorption behavior of fluorene and phenanthrene onto the DIM and the NIM was evaluated using the Langmuir, Freundlich, and Temkin isotherm models [36], as shown in Table 4. Based on the Langmuir equation, the maximum adsorption capacities of DIM were calculated to be 130.857 mg/g for fluorene and 453.030 mg/g for phenanthrene, which were markedly higher than those of NIM (65.047 mg/g for fluorene and 208.343 mg/g for phenanthrene). From these results, imprinting factors (IF)—defined as the ratio of the adsorption capacity of the DIM to that of the NIM—of approximately 2.01 for fluorene and 2.17 for phenanthrene were determined, confirming that the introduction of dummy templates successfully enhanced adsorption capacity and selectivity.

Analysis using the Freundlich model showed that favorable adsorption was indicated by 1/n values less than unity. For DIM, 1/n values of 0.530 for fluorene and 0.877 for phenanthrene were obtained, while NIM exhibited values of 0.293 for fluorene and 0.804 for phenanthrene. These results suggested that adsorption occurred on heterogeneous surfaces with varying binding energies, and that the heterogeneity of DIM was greater than that of NIM due to the presence of specific recognition cavities formed during imprinting.

The Freundlich constant (K_F_), which reflects the adsorption capacity and affinity in heterogeneous systems, was substantially higher in DIM than in NIM. For fluorene, the K value increased from 70.424 to 434.227, and for phenanthrene, from 142.833 to 1709.325, respectively. These values confirm the enhanced affinity and adsorption capacity of DIM, particularly for phenanthrene.

The Temkin isotherm analysis also produced high correlation coefficients (R^2^ = 0.9798–0.9931), confirming that the model adequately described the adsorption process. The constant ln A, which is related to the Temkin binding equilibrium constant, was found to be higher for DIM (5.603 for fluorene; 5.122 for phenanthrene) than for NIM (5.051 for fluorene; 2.891 for phenanthrene). These values indicated that the binding affinity of DIM toward the target analytes was significantly enhanced compared to NIM, reflecting the presence of specific recognition sites generated during the imprinting process. Thus, the adsorption of fluorene and phenanthrene on DIM was governed not only by sorbent–sorbate interactions but also by the increased affinity associated with the imprinted cavities.

The higher adsorption capacity of DIM toward phenanthrene was attributed to its structural planarity, consisting of three fused six-membered rings arranged in a flat conformation. In contrast, fluorene contains a central five-membered ring that induces a bent molecular geometry, thereby reducing the efficiency of π–π stacking interactions. The planarity of phenanthrene enables stronger π–π interactions with the aromatic moieties of the polymer matrix, particularly those derived from styrene, thus contributing to its higher adsorption performance. Anthrone, used as the dummy template, also exhibits a relatively planar structure due to its fused aromatic rings and nearly planar cyclohexanone group. This structural similarity between anthrone and phenanthrene enhances the imprinting effect, resulting in better recognition and higher adsorption capacity for phenanthrene compared to fluorene, which lacks such planarity.

### 2.8. Application to Simulated River Water Samples

**Figure 7:**
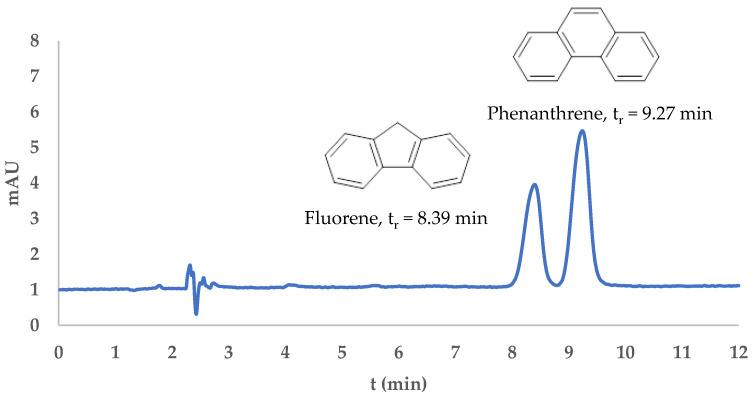
HPLC chromatogram of a standard mixture of fluorene and phenanthrene (100 ppb in acetonitrile).

**Figure 8:**
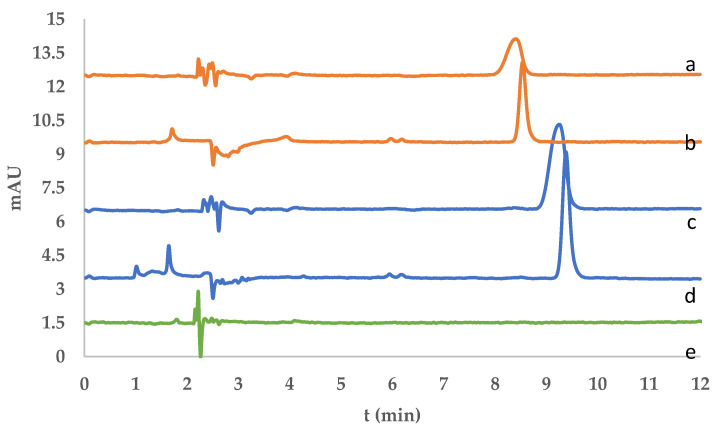
Overlay chromatograms of river water samples: (a) desorbed fluorene, (b) fluorene spiked sample before extraction and (c) desorbed phenanthrene, (d) phenanthrene spiked sample before extraction and (e) blank acetonitrile.

The analysis was performed using HPLC. Fluorene and phenanthrene were eluted at retention times of 8.39 min and 9.27 min, respectively, with a resolution value of 1.590, indicating satisfactory peak separation and the effectiveness of the method, as shown in Figure 7. The chromatographic profiles confirmed the applicability of the developed DIM-based dispersive solid-phase extraction (DSPE) method for the selective isolation and analysis of fluorene and phenanthrene. After SPE treatment, both analytes were successfully desorbed and detected, as evidenced by the appearance of well-resolved peaks in the desorption chromatograms (Figure 8).

In addition to these chromatographic results, the recovery performance of the DIM was evaluated for fluorene and phenanthrene at spiking levels of 50, 100, and 150 ppb. As shown in Table 5, the DIM sorbent exhibited satisfactory recoveries for both fluorene (86.61–112.42%) and phenanthrene (88.83–92.40%), all within the accepted range of 80–120%. The low RSD values (1.34–4.70%) confirmed good precision. The second use on the same sorbent, a marked decrease in fluorene recovery was observed, falling below the acceptable limit, likely due to incomplete desorption and fouling of the imprinting sites. In contrast, phenanthrene maintained recoveries within the acceptable range, albeit slightly reduced. Therefore, the membrane is recommended for single use when targeting fluorene, whereas limited reuse may be feasible for phenanthrene.

Table 6 summarizes the imprinting factor of MIPs reported in previous studies in comparison with the present anthrone-based DIM. Conventional MIPs prepared by sol–gel and bulk polymerization exhibited relatively low adsorption capacities, typically in the range of 0.1110–0.1763 mg/g for sol–gel systems and up to 1.27 mg/g for bulk MIPs [37,38,39]. In contrast, the present study achieved markedly higher adsorption capacities of 130.857 mg/g for fluorene and 453.030 mg/g for phenanthrene. Although the imprinting factors obtained (2.01 for fluorene and 2.17 for phenanthrene) are comparable to or lower than those of some bulk systems, the significant enhancement in adsorption capacity highlights the superior performance of the electrospun PVDF-supported DIM. This improvement is attributed to the nanofibrous structure, which provides a high surface area and uniformly distributed binding sites, thereby facilitating greater analyte accessibility and efficient adsorption.

## 3. Materials and Methods

### 3.1. Computational Study

The computational procedure applied the GAFF force field to evaluate the interactions between anthrone, fluorene, phenanthrene, and styrene. Three-dimensional molecular structures were constructed and optimized using the steepest descent algorithm. Binary complexes were obtained from random docking orientations to represent diverse binding conformations. The optimized geometries from GAFF (molecular dynamics) were then used to calculate binding energies (ΔE) via single-point energy evaluation at the PBEh-3c/DFT level with the def2-mSVP basis set, where ΔE was determined according to Equation (1), [26,27,28,29,30,31,32,33]:

(1) $$∆E=E_{complex}-\left(E_{monomer}+E_{template}\right)$$

where E_complex_ is the total energy of the optimized monomer–template complex, E_monomer_ is the total energy of the optimized monomer, and E_template_ is the total energy of the optimized template molecule. All energy values are expressed in kJ.mol^−1^. DFT calculations were performed using Orca v4.2.1 on a personal computer equipped with an AMD Ryzen 5 4500U processor (2.38 GHz CPU), 8 GB memory, and a 1 TB hard disk, running on the Windows 10 operating system.

### 3.2. Chemicals and Reagents

All reagents and solvents used in this study were of analytical or HPLC grade and were obtained from Merck (Darmstadt, Germany). Poly(vinylidene fluoride) (PVDF) with an average molecular weight of 180,000 g/mol was used as the base polymer for membrane fabrication. Anthrone was used as a dummy template, while styrene served as the functional monomer. Ethylene glycol dimethacrylate (EGDMA) was used as the cross-linking agent, and benzoyl peroxide (BPO) was used as the radical initiator. Acetonitrile (ACN) was used both as a porogenic solvent in the polymerization process and as the mobile phase in HPLC analysis. N,N-Dimethylformamide (DMF) was used as the solvent for the electrospinning process. Fluorene and phenanthrene were used as target analytes in adsorption studies. AquaDM was used in all aqueous preparations and adsorption experiments.

### 3.3. Synthesis of PVDF Membrane via Electrospinning

The PVDF membrane was prepared using the electrospinning technique. A 20% (*w*/*v*) PVDF solution was prepared by dissolving PVDF powder in DMF. The solution was stirred using a magnetic stirrer at 60 °C for 3 h until complete homogenization was achieved. The homogeneous solution was then transferred into a 3 mL polyethylene syringe equipped with a stainless-steel needle with an internal diameter of 0.69 mm.

Electrospinning was carried out at room temperature using a Nachriebe 601 electrospinning apparatus (Integrated Laboratory of Materials and Instrumentation, Department of Physics, Institut Teknologi Bandung (ITB), Bandung, Indonesia). The solution was delivered at a flow rate of 0.5 mL/h using a syringe pump. A high-voltage power supply was used to apply an electrical voltage of 16 kV between the needle tip and the collector. The distance between the needle tip and the collector was maintained at 18 cm. The resulting nanofibrous PVDF membrane was collected and dried at room temperature prior to further use.

### 3.4. Synthesis of DIM

The DIM was prepared through an in situ polymerization process. A total of 250 mg of electrospun PVDF membrane was immersed in a pre-polymerization solution containing 0.5 mmol of anthrone (dummy template), 1 mmol of functional monomer (styrene), 20 mmol of cross-linker (EGDMA), and 250 mg of BPO in 50 mL of acetonitrile, which acted as the porogenic solvent.

**Figure 9:**
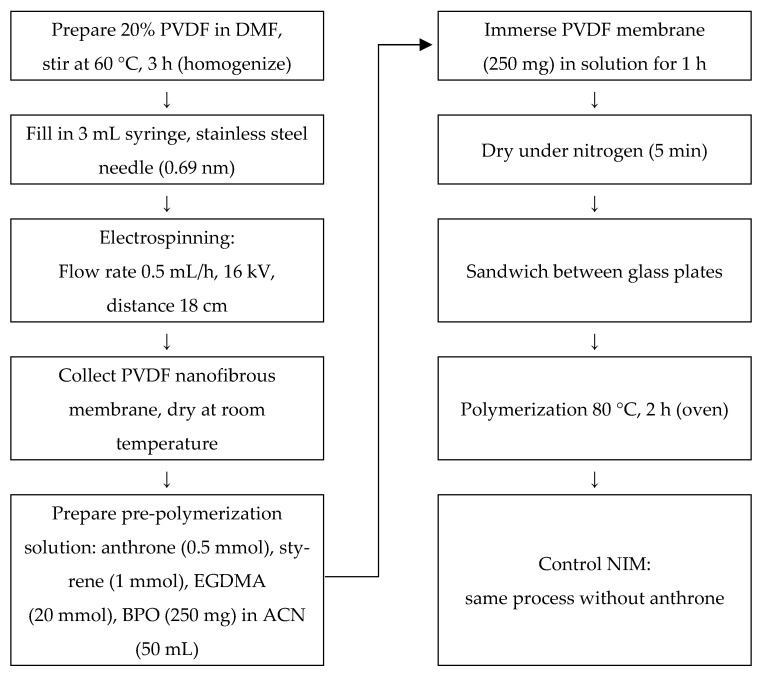
Schematic illustration of the preparation of DIM.

The immersion was carried out for 1 h at room temperature to ensure uniform infiltration of the pre-polymerization solution into the PVDF matrix. After soaking, the membrane was gently dried under a nitrogen gas stream for 5 min. It was then sandwiched between two glass plates to maintain its shape and structural integrity during polymerization. The polymerization process was conducted in an oven at 80 °C for 2 h. As a control, a NIM was synthesized using the same procedure but without the addition of the template molecule (anthrone). The overall preparation steps are illustrated in Figure 9.

### 3.5. Leaching of the DIM

A total of 50 mg DIM was immersed in 10 mL of acetonitrile for template removal. The leaching process was repeated until anthrone was no longer detected in the filtrate, as confirmed by UV–Vis spectrophotometer (Agilent 8453 G1103A, Agilent Technologies, Santa Clara, CA, USA), which was used to monitor anthrone leaching in each washing fraction.

### 3.6. Characterization Techniques

Material characterization was carried out to evaluate the structural, morphological, and physicochemical properties of the synthesized DIM and NIM polymers. FTIR spectra were recorded using a Shimadzu Prestige 21 spectrometer to identify the functional groups present in the samples. Surface morphology was by SEM (Hitachi SU 3500, Hitachi High-Technologies Corporation, Krefeld, Germany). Specific surface area, pore diameter, and pore volume were determined by BET method (BELSORP-miniX 332 version 1.1.7.0). BELSORP-miniX 332 version 1.1.7.0 Thermal stability of the materials was evaluated by TGA (NETZSCH STA 449 F1, NETZSCH-Gerätebau GmbH, Selb, Germany). All characterizations were performed on DIM after leaching, with NIM as a control.

### 3.7. Adsorption Performance

Stock solutions of fluorene and phenanthrene (1000 ppm) were prepared by dissolving 10 mg of each compound in acetonitrile. The compounds were accurately weighed using an analytical balance and transferred into separate 10 mL volumetric flasks. A small amount of acetonitrile was added to facilitate dissolution, followed by dilution to the mark with the same solvent. The resulting stock solutions were stored in tightly sealed amber glass bottles and protected from light to prevent photodegradation.

For adsorption experiments, aqueous test solutions with concentrations ranging from 0.4 to 1.4 ppm were prepared by diluting the stock solutions with deionized water. The appropriate volume of the 1000 ppm stock was pipetted (e.g., 200 µL for 0.4 ppm and 700 µL for 1.4 ppm) into a 500 mL volumetric flask, followed by dilution to the mark with deionized water. The solutions were mixed thoroughly by gentle shaking to ensure homogeneity.

Adsorption isotherm experiments were conducted to evaluate the adsorption performance of the DIM compared to the NIM. For each test, 2.5 mg of DIM or NIM was added to 200 mL of fluorene or phenanthrene solution at varying concentrations. The concentration range for fluorene was 0.4–1.4 ppm, while for phenanthrene it was 0.4–1.2 ppm. The mixtures were placed in sealed glass vessels and agitated gently at room temperature for 2 h to reach adsorption equilibrium.

After the equilibration period, the membranes were separated from the solution by decantation. The resulting supernatant was then filtered through a membrane filter to remove any remaining particulates prior to high-performance liquid chromatography (HPLC, Infinity Agilent Tech. 1260 series) analysis, equipped with a C-18 reversed-phase column (Fortis UniverSil, 5 µm particle size, 250 mm × 4.6 mm), a mobile phase consisting of acetonitrile and water (70:30, *v*/*v*) at a flow rate of 1 mL/min and a detection wavelength of 254 nm.

The adsorption capacity (q_e_, mg/g) at equilibrium was calculated using Equation (2).

(2) $$q_{e}=\frac{\left(C_{0}-C_{e}\right)\times V}{m},$$

where C_0_ and C_e_ are the initial and equilibrium concentrations (ppm), V is the solution volume (L), and m is the adsorbent mass (g).

The adsorption capacity data obtained from DIP and NIP were subsequently fitted to three linearized isotherm models: the Langmuir model, the Freundlich model, and the Temkin model, corresponding to Equation (3), Equation (4), and Equation (5), respectively. This fitting process was carried out to determine the most suitable adsorption isotherm for describing the interaction between the sorbents and the target analytes, as well as to estimate the relevant adsorption parameters [36].

(3) $$\frac{1}{q_{e}}=\frac{1}{q_{max}\times K_{L}\times C_{e}}+\frac{1}{q_{max}},$$

(4) $$\log q_{e}=\log K_{F}+\frac{1}{n}\log C_{e},$$

(5) $$q_{e}=B\ln A+B\ln C_{e},$$

where q_e_ (mg/g) is the adsorption capacity at equilibrium, C_e_ (mg/L) is the equilibrium concentration (ppm), q_max_ (mg/g) is the maximum adsorption capacity, and K_L_ (L/mg) is the Langmuir adsorption constant. K_F_ [(mg.g^−1^)(L.mg^−1^)^1/n^] is the Freundlich adsorption capacity constant, and 1/n is the surface heterogeneity factor. In the Temkin model, B (J/mol) is related to the heat of adsorption, and A (L/g) is the Temkin binding equilibrium constant.

### 3.8. Application to Simulated River Water Samples

Prior to use, river water samples were filtered through Whatman No. 42 filter paper to remove suspended solids. Simulated water samples were prepared by spiking the filtered river water with fluorene and phenanthrene at concentrations of 50, 100, and 150 ppb, using a 100 ppm stock solution. The spiking process was conducted in 50 mL volumetric flasks. Specifically, 25 µL, 50 µL, and 75 µL of the stock solution were added to separate flasks to obtain the respective concentrations. After the addition of the stock solutions, each flask was filled to the mark with filtered river water, sealed, and gently shaken to ensure uniform mixing. All spiked solutions were freshly prepared immediately prior to analysis.

The analysis of fluorene and phenanthrene in these samples was carried out using a batch adsorption procedure. Prior to adsorption, 50 mg of the DIM was pre-conditioned by soaking in 10 mL of acetonitrile for 30 min to activate the surface and remove any loosely bound residues. Following pre-conditioning, the membrane was transferred into 10 mL of the spiked sample solution and allowed to adsorb the analytes under gentle agitation for 15 min at room temperature. After adsorption, the membrane was briefly rinsed with 10 mL of ultrapure water to eliminate non-specifically bound hydrophilic components. Subsequently, desorption of the retained analytes was performed by immersing the membrane in 10 mL of acetonitrile for 15 min, allowing the bound fluorene and phenanthrene to be effectively released into the solvent phase.

**Figure 10:**
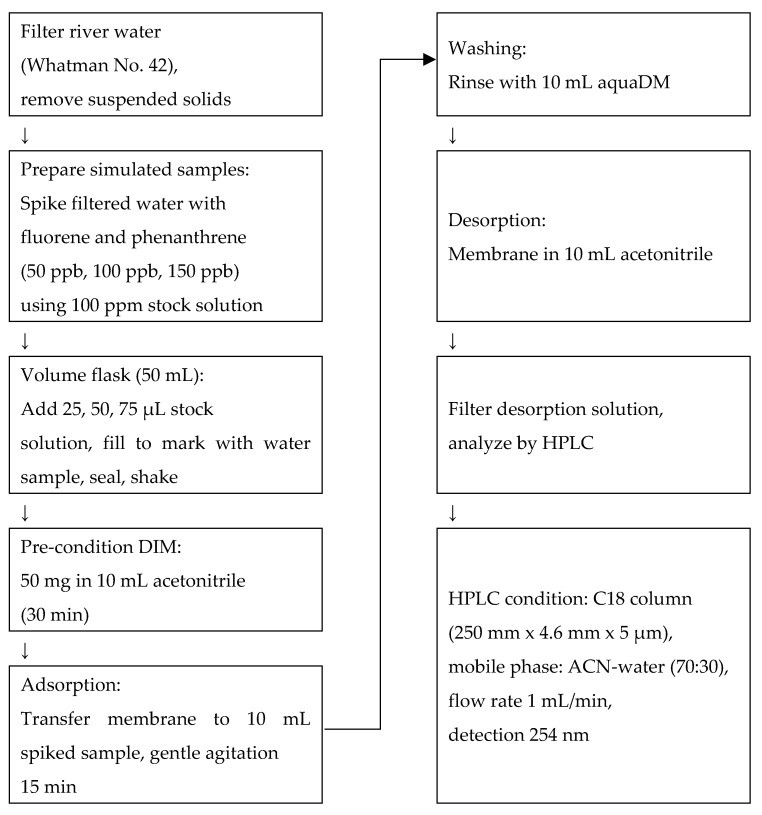
Schematic illustration of the application of DIM in DSPE.

The resulting desorption solution was filtered to remove particulates and subjected to analysis by high-performance liquid chromatography (HPLC). The HPLC system was equipped with a C-18 reversed-phase column (Fortis UniverSil, 5 µm particle size, 250 mm × 4.6 mm). The mobile phase consisted of acetonitrile and water (70:30, *v*/*v*), delivered at a flow rate of 1.0 mL/min, and detection was carried out at 254 nm. This analytical setup, as shown in Figure 10, enabled efficient separation and quantification of fluorene and phenanthrene following the membrane-based extraction procedure.

### 3.9. Novelty of Present Study

The results obtained in this study highlight several distinctive features of the proposed approach compared with conventional MIP systems in bulk or particle form. Firstly, the use of a dummy template (anthrone) instead of the real analyte avoids the risk of target leakage, thereby improving safety and analytical reliability. Secondly, the development of a DIM supported on electrospun PVDF fibers combines high surface area and well-distributed imprinted sites, resulting in enhanced adsorption capacity and better accessibility compared to bulk MIP structures. Furthermore, the membrane form is thin, flexible, and easy to handle, eliminating the need for grinding, sieving, and packing into cartridges as required by conventional bulk MIPs. Finally, the combination of electrospinning with in situ polymerization for dummy imprinting represents a novel fabrication strategy that improves selectivity and usability.

Beyond the methodological novelty, the most important contribution of this work lies in its direct applicability to environmental monitoring of PAHs. The ability of the DIM to selectively capture fluorene and phenanthrene, two representative low-molecular-weight PAHs frequently found in polluted rivers, demonstrates its relevance for water quality assessment. By integrating safety (dummy template), structural innovation (semi-IPN PVDF membrane), and proven analytical performance (high adsorption capacity and acceptable recoveries), this study offers not only a methodological advancement but also a practical tool for routine monitoring of hazardous PAHs in aquatic environments. These unique aspects collectively highlight the originality and practical advantages of the present study over traditional MIP approaches, as shown in Table 7.

## 4. Conclusions

The anthrone-based DIM was successfully synthesized via an in situ polymerization method based on a semi-IPN, using anthrone as a dummy template to selectively adsorb low-molecular-weight PAHs, particularly fluorene and phenanthrene. Characterization by FTIR, TGA, SEM, and BET confirmed the formation of specific recognition sites and porous structures after template removal. HPLC analysis of simulated samples validated the selectivity and recovery performance of the DIM sorbent, showing clear chromatographic separation of both analytes with acceptable recovery values. Overall, the anthrone-based DIM demonstrated high potential as a selective sorbent for the simultaneous extraction and analysis of fluorene and phenanthrene in environmental applications. Future studies could extend this strategy to other PAHs and real environmental samples, as well as explore scale-up for practical monitoring applications.

## Figures and Tables

**Table 1:** HOMO–LUMO Energy Levels and Bandgap of Styrene Complexes.

Complex	HOMO (eV)	LUMO (eV)	Bandgap (eV)	ΔE (kJ/mol)
anthrone–styrene 1:1	−7.4631	−1.2244	6.2387	−19.20
fluorene–styrene 1:1	−6.8554	−0.3138	6.5416	−17.88
phenanthrene–styrene 1:1	−6.6280	−0.6890	5.9390	−18.49

**Table 2:** PBEh-3c Estimation of Optimal Template-to-Monomer Ratios.

Ratio	HOMO (eV)	LUMO (eV)	Bandgap (eV)	ΔE (kJ/mol)
anthrone–styrene 1:1	−7.4631	−1.2244	6.2387	−19.20
anthrone–styrene 1:2	−7.0627	−1.1186	5.9441	−54.25
anthrone–styrene 1:3	−7.0963	−1.1201	5.9762	−38.75

**Table 3:** BET Surface Area and BJH Desorption Pore Characteristics of DIM and NIMs.

Sorbent	Surface Area (m^2^/g)	Pore Volume (cc/g)	Pore Radius (nm)
DIM	4.662	0.045	18.521
NIM	0.447	0.015	34.237

**Table 4:** Adsorption isotherm parameters of DIM and NIM for fluorene (FLU) and phenanthrene (PHEN) at room temperature (25 °C).

Isotherm Models	Parameter	NIM-FLU	DIM-FLU	NIM-PHEN	DIM-PHEN
Langmuir	slope	0.0013	0.0002	0.0041	0.0003
1/C_e_ vs. 1/q_e_	intercept	0.0154	0.0076	0.0048	0.0022
	R^2^	0.9967	0.9912	0.9999	0.9999
	q_max_ (mg/g)	65.047	130.857	208.343	453.030
	K (L/mg)	12.040	38.613	1.180	7.032
Freundlich	slope	0.2932	0.5302	0.8041	0.8772
log C_e_	intercept	1.8477	2.6377	2.1548	3.2328
vs log q_e_	R^2^	0.9786	0.9998	0.9990	0.9997
	K_F_ (mg/g)(L/mg)^1/n^	70.424	434.227	142.833	1709.325
	1/n	0.293	0.530	0.804	0.877
Temkin	slope	13.0245	33.6975	32.6321	49.1895
ln C_e_ vs. q_e_	intercept	65.7853	188.8070	94.3444	251.9261
	R^2^	0.9931	0.9841	0.9860	0.9798
	ln A	5.051	5.603	2.891	5.122
	B (J/mol)	13.024	33.697	32.632	49.190

**Table 5:** Recovery of fluorene and phenanthrene using DIM sorbent on first use and second use.

Analyte	Spike (ppb)	First Use		Second Use	
		% Recovery	RSD (%)	% Recovery	RSD (%)
fluorene	50	112.42	4.06	29.51	3.25
	100	86.61	2.60	25.69	2.74
	150	104.12	1.34	34.42	1.35
phenanthrene	50	88.83	4.70	88.30	3.68
	100	92.40	4.32	87.16	3.07
	150	91.06	3.20	90.61	2.60

**Table 6:** Comparison of Imprinting Factors of DIM with Several MIPs for Fluorene and Phenanthrene Analysis.

Template	Components	Materials	Synthesis Approach	Target Analyte	q_e_ (mg/g)	IF	Ref
16 PAHs	PTMS, TEOS	MIP	Sol–gel	Fluorene	0.1110	1.92	[37]
				Phenanthrene	0.1763	1.91	
Pyrene	MAA, 4-VP, EGDMA	MIP	Bulk polymerization	Phenanthrene	0.37	2.47	[38]
16 PAHs	MAA, EGDMA	MIP	Bulk polymerization	Fluorene	1.22	9.38	[39]
				Phenanthrene	1.27	5.29	
Anthrone	Styrene, EGDMA, PVDF	DIM	Electrospinning, semi-IPN	Fluorene	130.857	2.01	Present Study
				Phenanthrene	453.030	2.17	

**Table 7:** Novelty of present study.

Feature/Parameter	Anthrone-Based DIM	Conventional MIP (Bulk or Particles)
Template Type	Dummy template (anthrone) which avoids target leakage	Real template, with risk of template leakage
Selectivity for PAHs	High, due to the presence of well-defined imprinted cavities	High, but limited by accessibility of binding sites
Adsorption capacity	High, attributed to the large surface area of electrospun PVDF and uniformly distributed imprinting sites	Moderate, due to bulk structure
Materials	Membrane, thin, flexible, and easy to handle	Powder form, requiring grinding, sieving, and cartridge packing
Preparation Complexity	Moderate, involving electrospinning and in situ polymerization	High, involving bulk polymerization followed by grinding and sieving
Novelty aspect	Integration of dummy imprinting with electrospun PVDF support, providing improved selectivity, adsorption efficiency, and safety	Limited to conventional imprinting on powder without membrane support

## Data Availability

All data supporting the findings of this study are contained within the article.

## Abbreviations

The following abbreviations are used in this manuscript:

ACN	Acetonitrile
BJH	Barrett–Joyner–Halenda
BET	Brunauer–Emmett–Teller
DFT	Density Functional Theory
DIM	Dummy Molecularly Imprinted Membrane
DSPE	DIM-based Dispersive Solid-Phase Extraction
FLU	Fluorene
FTIR	Fourier-Transform Infrared Spectroscopy
GAFF	Generalized Amber Force Field
HOMO	Highest Occupied Molecular Orbital
HPLC	High-Performance Liquid Chromatography
LUMO	Lowest Unoccupied Molecular Orbital
IF	Imprinting Factors
MIP	Molecularly Imprinted Polymer
NIM	Non-Imprinted Membrane
PHEN	Phenanthrene
PVDF	Poly(vinylidene Fluoride)
PAHs	Polycyclic Aromatic Hydrocarbons
SEM	Scanning Electron Microscopy
semi-IPN	Semi-Interpenetrating Polymer Network
TGA	Thermogravimetric Analysis
